# TLR4 Targeting as a Promising Therapeutic Strategy for Alzheimer Disease Treatment

**DOI:** 10.3389/fnins.2020.602508

**Published:** 2020-12-18

**Authors:** Yongji Zhou, Yanxing Chen, Congcong Xu, Hao Zhang, Caixiu Lin

**Affiliations:** ^1^Department of Neurology, Affiliated Hangzhou First People’s Hospital, Zhejiang University School of Medicine, Hangzhou, China; ^2^Department of Neurology, The Second Affiliated Hospital, Zhejiang University School of Medicine, Hangzhou, China; ^3^The First Affiliated Hospital, Zhejiang University School of Medicine, Hangzhou, China

**Keywords:** Alzheimer’s disease, toll-like receptor 4, neuroinflammation, microglia, therapeutic target

## Abstract

Alzheimer disease (AD) is a devastating neurodegenerative disorder characterized by extracellular accumulation of amyloid-beta and formation of intracellular neurofibrillary tangles. Microglia activation and neuroinflammation play important roles in the pathogenesis of AD; Toll-like receptor 4 (TLR4)—a key component of the innate immune system—in microglia is also thought to be involved based on the observed association between TLR gene polymorphisms and AD risk. TLR4 has been shown to exert both detrimental and beneficial effects on AD-related pathologies. In preclinical models, experimental manipulations targeting TLR4 were shown to improve learning and memory, which was related to inhibition of pro-inflammatory cytokine release and reduction of oxidative stress. In this review, we summarize the key evidence supporting TLR4 as a promising therapeutic target in AD treatment.

## Introduction

Alzheimer disease (AD) is a progressive and irreversible neurodegenerative disease that mainly manifests as memory loss and cognitive deterioration. Around 50 million people worldwide are afflicted with dementia, and this number is expected to exceed 131 million by 2050 with the aging of the global population (Alzheimer’s Association, 2019). AD is categorized into sporadic and familial forms. Sporadic AD, which is also known as late-onset AD (LOAD), accounts for 90% of all AD cases and mainly occurs in individuals over the age of 65 years. Familial AD has an earlier onset and is hereditary, with the genes encoding Aβ precursor protein (APP), presenilin 1 (PSEN1), and PSEN2 identified as causative genes (Bertram et al., 2010). However, the etiology of sporadic AD is not fully understood. The pathologic hallmarks of AD include extraneuronal accumulation of amyloid-beta (Aβ) plaques and intraneuronal aggregation of neurofibrillary tangles composed of tau protein (Querfurth and LaFerla, 2010), which are thought to be the major drivers of the disease. However, clinical trials of therapeutics targeting Aβ or tau aggregation have not yielded promising results (Castellani and Perry, 2012).

Neuroinflammation is a prominent pathologic feature of AD. Genes related to immunity identified in genome-wide association studies have been linked to the risk of sporadic AD, including those encoding complement receptor 1 (CR1), cluster of differentiation 33 (CD33), and triggering receptor expressed on myeloid cells 2 (TREM2) (Karch and Goate, 2015). Elevated levels of inflammatory cytokines and chemokines have been detected in postmortem brains of AD patients (Cacabelos et al., 1991; Grammas and Ovase, 2001). Thus, the innate immune response plays a role in the development of AD (Jevtic et al., 2017). Microglia are brain-resident macrophages and the most important innate immune cells in the central nervous system (CNS). In the brain of AD patients, microglia are present around senile plaques in an activated state, implying that they are involved in disease pathogenesis (Hansen et al., 2018). Activated microglia load is associated with the upregulation of various cell surface receptors and pro-inflammatory molecules in AD. Toll-like receptors (TLRs) of the innate immune system function as pattern recognition receptors (PRRs) and have been implicated in AD (McGeer et al., 1987; Su et al., 2016). As the first line of defense against pathogens, TLRs sense pathogen-associated molecular patterns and danger-associated molecular patterns (DAMPs) including Aβ.

To date, 10 functional human TLRs (TLR1–10) and 12 mouse TLRs (TLR1–9, 11–13) have been identified (Kawasaki and Kawai, 2014). Human microglia express TLRs 1–9 (Bsibsi et al., 2002). TLR4 is expressed on the surface of microglia and plays a critical role in neuroinflammation by binding to Aβ fibrils. In this review, we discuss the involvement of TLR4 in AD and evidence from animal models supporting TLR4 as a potential therapeutic target in AD treatment.

## Toll-Like Receptor 4 Signaling

TLRs are type I transmembrane proteins that consist of an extracellular leucine-rich repeat ligand-binding domain, single membrane-spanning helix, and intracellular signaling Toll/interleukin-1 receptor (TIR) domain (Moresco et al., 2011). Following ligand binding, TLRs undergo dimerization or oligomerization and recruit TIR domain adaptors, resulting in the synthesis and release of pro- and anti-inflammatory molecules. Four adaptor proteins are known to be activated by TLRs including myeloid differentiation primary response protein 88 (MyD88), MyD88 adaptor-like/TIR domain-containing adaptor molecule (Mal/TIRAP), TIR domain-containing adaptor protein-inducing interferon-β [TRIF; also known as TIR domain-containing adaptor molecule 1 (TICAM1)], and TRIF-related adaptor molecule [TRAM; also known as TIR domain-containing adaptor molecule 2 (TICAM2)] (Moresco et al., 2011).

**FIGURE 1:**
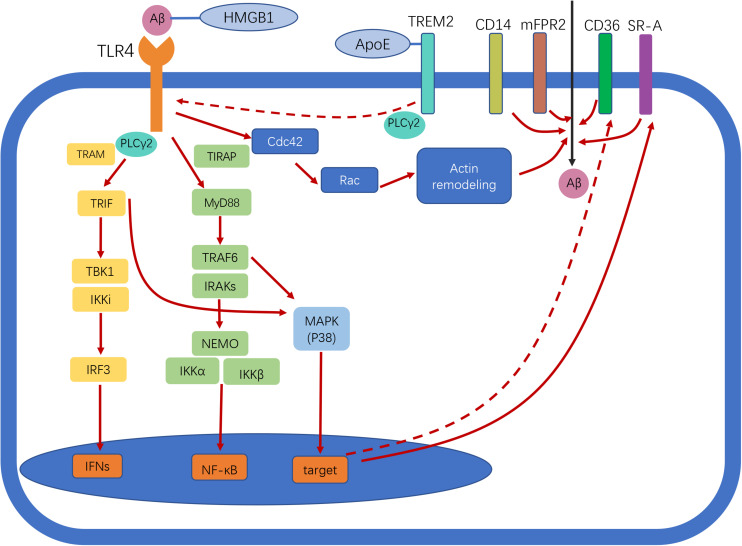
Toll-like receptor 4 (TLR4) signaling in Alzheimer disease (AD). Following ligand binding, TLR4 activates downstream signaling pathways through myeloid differentiation primary response protein 88 (Myd88)-dependent and -independent pathways, leading to nuclear factor-κB (NF-κB) and interferon-β (IFN-β) activation. TLR4 activation enhances amyloid-beta (Aβ) phagocytosis by microglia. Scavenger receptors (SRs), cluster of differentiation (CD)14, and murine formyl peptide receptor 2 (mFPR2) are involved in this process. The two different pathways involved in Aβ uptake by microglia are MyD88–p38–SR and the MyD88-independent Cdc42/Rac pathway. TLR4 activation reduces CD36 expression, thereby inhibiting CD36-mediated phagocytosis of Aβ. Triggering receptor expressed on myeloid cells 2 (TREM2) negatively regulates TLR-induced inflammatory cytokine production. TREM2 regulates TLR4/phospholipase C γ2 (PLCγ2)-dependent inflammatory signaling. TREM2 interacts with apolipoprotein E (ApoE) to affect phagocytosis of apoptotic neurons. High-mobility group box protein 1 (HMGB1) interacts with Aβ and inhibits Aβ phagocytosis by microglia via TLR4 signaling. Straight and dotted arrows represent activation and inhibition, respectively.

TLR4 is a member of the TLR family that specifically recognizes lipopolysaccharide (LPS), a glycolipid present in the outer membrane of most Gram-negative bacteria. LPS is typically composed of a hydrophobic domain, Lipid A (also known as an endotoxin), a non-repeating “core” oligosaccharide, and a hydrophilic polysaccharide (or O-antigen) (Raetz and Whitfield, 2002). The extracellular molecules MD-2 and CD14 are required for TLR4 to recognize and process LPS (Akira et al., 2006). Stimulation of TLR4 induces the activation of MyD88-dependent and -independent pathways. In the former, MyD88 mediates the activation of interleukin 1 (IL-1) receptor-associated kinases (IRAKs) and tumor necrosis factor (TNF) receptor-associated factor 6 (TRAF6), which is followed by activation of the inhibitor of nuclear factor kappaB (IκB) kinase complex (IKK complex) that includes IKK-α, IKK-β, and IKK-γ [also known as IKK1, IKK2, and nuclear factor-κB (NF-κB) essential modulator (NEMO), respectively] (Kawai and Akira, 2007). This pathway activates NF-κB, which leads to the transcription of genes encoding pro-inflammatory factors such as TNF-α and IL-1. TRAF6 activates the mitogen-activated protein kinase (MAPK) signaling pathway [which includes extracellular signal-regulated kinase (ERK), c-Jun N-terminal kinase (JNK), and p38], leading to an inflammatory response. In the MyD88-independent/TRIF-dependent pathway, TLR4 cooperates with TRIF to induce interferons (IFNs; e.g., IFN-α/β) and NF-κB (Kawai and Akira, 2007). The N- and C-terminal regions of TRIF have distinct functions with regard to the recruitment of downstream effectors. The N terminus recruits non-canonical IKKs, TANK-binding kinase 1 (TBK1) (also known as T2K or NAK), and IKK-inducible kinase (IKKi; also known as IKKε), which phosphorylate the C terminus of IFN regulatory factor 3 (IRF3) to induce the expression of target genes including IFN-β (Kawai and Akira, 2007). There is crosstalk between the TRIF-dependent and MyD88-dependent pathways in that the N terminus of TRIF recruits TRAF6 and induces NF-κB activation (Kawai and Akira, 2007). Moreover, the C terminus of TRIF interacts with receptor-interacting serine/threonine-protein kinase 1 (RIP1), which forms a complex with TRAF6 and transforming growth factor β-activated kinase 1 (TAK1) to induce the activation of NF-κB and MAPK (Kawai and Akira, 2007). Thus, the TRIF N terminus activates both IFN-β and NF-κB promoters, whereas the C terminus activates only the latter. TRAM links TLR4 and TRIF in the activation of the TRIF-dependent pathway, while TIRAP selectively induces the activation of the MyD88-dependent pathway downstream of TLR4 (Akira and Takeda, 2004). The TLR4 signaling pathway is summarized in Figure 1.

## Alzheimer Disease and Toll-Like Receptor 4

In the CNS, TLR4 is expressed in microglia, oligodendrocytes, and astrocytes (Bowman et al., 2003; Olson and Miller, 2004). TLR4 has also been detected in mammalian neurons, although at a very low level (Bsibsi et al., 2002). Here we focus on TLR4 in microglia, given their critical role as CNS immune cells.

Several single-nucleotide polymorphisms (SNPs) of TLR4 are linked to AD susceptibility. The Asp299Gly polymorphism of the *TLR4* gene was shown to attenuate the inflammatory response and is thought to protect against the development of sporadic AD (Minoretti et al., 2006). A minor allele (G) of rs4986790 was associated with a lower AD risk in an Italian cohort, which was attributed to reduced IL-1β production and release in preclinical-stage familial AD (FAD) cases (Miron et al., 2019). Rs4986790 A/G is a common missense mutation that is associated with elevated anti-inflammatory cytokine levels, which has a protective effect against neuroinflammation upon exposure to LPS. Several other SNPs of *TLR4* have been reported to confer neuroprotection in the Han Chinese population—i.e., rs10759930, rs1927914, rs1927911, rs12377632, rs2149356, rs7037117, and rs7045953, whereas rs11367 and rs1927907 increase the risk of AD (Wang et al., 2011; Chen et al., 2012; Yu et al., 2012). However, the precise function of most of these gene variants in AD has not been established.

The involvement of TLR4 in AD is supported by other lines of evidence. Firstly, TLR4 is upregulated in transgenic mice overexpressing APP. High TLR4 immunoreactivity was observed in glial cells surrounding plaques in postmortem brains of AD patients (Walter et al., 2007), and significantly higher levels of pro-inflammatory cytokines have been detected in the brains of APP/PS1 mice compared to TLR4-mutant APP/PS1 mice (Jin et al., 2008). Secondly, oligomeric and fibrillar Aβ peptide can induce TLR4-dependent microglia activation, which requires a trimolecular complex composed of TLR4, myeloid differentiation factor 2 (MD-2), and CD14 (Walter et al., 2007). Thirdly, supernatant from LPS-stimulated wild-type (WT) microglia caused more extensive neuronal death than that from TLR4-mutated microglia, implying that the release of neurotoxic products by microglia is TLR4-dependent (Walter et al., 2007). Finally, intracerebroventricular injection of Aβ induced an inflammatory response leading to neuronal death, synaptic loss, and cognitive impairment in WT mice but not in TLR4 knockout mice; moreover, a selective TLR4 receptor antagonist abolished Aβ oligomer-induced microglia activation and memory impairment, which was not observed in mice lacking TLR4 (Balducci et al., 2017).

The abovementioned findings strongly indicate that TLR4 activation is associated with the development of AD pathology and cognitive impairment. However, TLR4 may also play a neuroprotective role in AD. When cultured microglia were treated with LPS and then incubated with Aβ42 for 24 h, Aβ42 in the culture medium was reduced by ∼50%, indicating that TLR4 increased Aβ clearance (Tahara et al., 2006). This also suggests that microglia can be activated via TLR4 signaling at the early stage of β-amyloidosis to inhibit Aβ deposition, thereby protecting neurons from Aβ-mediated neurotoxicity (Song et al., 2011). However, as the disease progresses, continuous exposure of microglia to Aβ attenuates the response by TLR4, and activated microglia become incapable of clearing Aβ deposits (Go et al., 2016). This immune tolerance is abolished by early low-level stimulation of microglia with TLR4 agonists such as LPS or monophosphoryl lipid A (MPL), which were shown to restore long-term potentiation that was impaired by Aβ and improve spatial and working memory in an AD rat model (Pourbadie et al., 2018). The underlying mechanism may involve TRIF-dependent signaling. Pretreatment with LPS or MPL was also found to enhance the expression of the neuroprotective cytokine IFN-β both *in vivo* and *in vitro* (Yousefi et al., 2019). MPL, a detoxified derivative of LPS, activates TLR4 to trigger the inflammatory response. The pyrogenicity of MPL is at least 100-fold lower than that of LPS, although in terms of most other immunomodulatory properties, the two molecules are comparable. In APP/PS1 mice, MPL administration induced actin remodeling and upregulation of scavenger receptor A (SR-A), which are essential for phagocytosis of extracellular materials such as Aβ; this resulted in marked reductions in the number and size of Aβ deposits and amount of soluble Aβ and improvements in cognitive function. In contrast to the strong phagocytic response, MPL only weakly induces inflammation. Importantly, repeated administration of MPL did not lead to immune tolerance, indicating that MPL is an effective yet safe drug for AD treatment (Michaud et al., 2013). Collectively, the existing evidence indicates that Aβ clearance is mediated by TLR4 activation and is achieved through enhanced phagocytosis. Thus, appropriate activation of TLR4 may inhibit AD progression by promoting Aβ clearance without inducing harmful neuroinflammation.

Phagocytosis of Aβ by microglia upon TLR signaling is mediated by several receptors, including SRs, CD14, and murine formyl peptide receptor 2 (mFPR2). Chemical blockers of these receptors have been shown to partially inhibit LPS-induced microglia uptake of Aβ, which is mainly mediated by the G protein-coupled receptor mFPR2. Pertussis toxin, a G protein receptor deactivator, reduced Aβ uptake by microglia by > 95% (Tahara et al., 2006). LPS-induced upregulation of mFPR2 in microglia may depend on activation of MAPK p38 and NF-κB. Activation of TLR signaling was shown to increase the expression of SR-A *via* the MyD88, IRAK4, and p38 signaling pathways, leading to significant enhancement of phagocytosis by macrophages/monocytes (Doyle et al., 2004). CD14 participates not only in LPS-induced internalization of TLR4 but also in the phagocytosis of Aβ42 fibrils in a clathrin-dependent manner (Fujikura et al., 2019). Additionally, actin filament assembly and dynamic rearrangement of the actin cytoskeleton are required for Aβ uptake. Small GTPases (e.g., Rac and Cdc42) are activated in response to LPS-induced actin assembly during phagocytosis, a process that does not rely on MyD88–p38 signaling (Kong and Ge, 2008). Thus, it is possible that LPS/TLR4-induced phagocytosis is mediated via two distinct pathways—namely, MyD88–p38–SR and MyD88-independent Cdc42/Rac pathways (Kong and Ge, 2008). However, conflicting findings have also been reported—for example, activation of TLR4 reduced the expression of CD36, a cell-surface SR, along with Aβ42 phagocytosis (Li et al., 2015). The possible mechanisms underlying the function(s) of TLR4 in AD are shown in Figure 1.

## Triggering Receptor Expressed on Myeloid Cells 2 and Toll-Like Receptor 4

Various mechanisms negatively regulate TLR4-driven inflammatory responses. TREM2, a transmembrane receptor belonging to the TREM family, is expressed on the surface of many myeloid cells including microglia, monocytes, macrophages, and dendritic cells. TREM2 is considered as a critical innate immune receptor of microglia that not only regulates biosynthetic metabolism, proliferation, survival, and cytokine release in these cells but also exerts a protective effect against Aβ pathology (Zhong et al., 2019). Polymorphism of the *TREM2* gene has been linked to a higher risk of developing LOAD (Guerreiro et al., 2013). During AD progression, homeostatic microglia switch to a disease-associated microglia (DAM) phenotype and prevent neurodegeneration (Keren-Shaul et al., 2017). Single-cell RNA sequencing analysis has revealed that TREM2 is critical for the second step of DAM activation (Keren-Shaul et al., 2017). Overexpression of TREM2 reduced the level of pro-inflammatory cytokines in microglia, whereas TREM2-activating antibody induced significant increases in both pro-inflammatory [IL-1β, TNF-α, C-C motif chemokine ligand (CCL)2, C-X-C motif chemokine ligand (CXCL)10, Gata3, Rorc] and anti-inflammatory (YM1 and IL1Rn) marker expression in 5 × FAD mice (Long et al., 2019; Price et al., 2020). Thus, TREM2 has complex functions in AD. TREM2 reduced the level of TLR4, resulting in decreased TLR-induced inflammatory cytokine production in dendritic cells (Ito and Hamerman, 2012). Overexpression of TREM2 decreased the level of TLR4, whereas *TREM2* gene silencing had the opposite effect (Long et al., 2019). Corresponding changes in downstream effectors of TLR4 (ERK, P38, and P65) and pro-inflammatory cytokines (IL-6, IL-1β, and TNF-α) were also observed in microglia overexpressing TREM2 (Long et al., 2019). Phospholipase C γ2 (PLCγ2) is an intracellular enzyme that cleaves the membrane phospholipid phosphatidylinositol-4,5-bisphosphate (PIP2); variants of the *PLC*γ*2* gene have been linked to AD (Sims et al., 2017). It was recently demonstrated that PLCγ2 mediates diverse functions of microglia through various upstream signaling molecules (e.g., TREM2 vs. TLR ligands). TREM2 was shown to attenuate the PLCγ2-mediated inflammatory response, and TLR4/PLCγ2-dependent inflammatory signaling was amplified in the absence of TREM2 (*TREM2* knockout) (Andreone et al., 2020). The brains of APP/PS1 mice were found to have higher levels of TLR4 and TREM2; after treatment with LPS, TLR4 was persistently upregulated in APP/PS1 mice, whereas the level of TREM2 was markedly reduced, suggesting that TREM2 has a negative modulatory effect on inflammation but that this is subjugated to the TLR4-induced response (Zhou et al., 2019). However, further study is needed to clarify the precise relationship between TREM2 and TLR4 in the context of AD.

## Apolipoprotein E and Toll-Like Receptor 4

Apolipoprotein E (ApoE) is a lipid-binding protein and 299 amino acids long. The three isoforms, ApoE2, ApoE3, and ApoE4, differ at positions 112 and 158 (Hatters et al., 2006). In addition to the well-known function of lipoprotein clearance, ApoE is also involved in inflammation modulation (Shi and Holtzman, 2018). When exposed to LPS intravenously, human subjects carrying *ApoE-*ε*4* genotype presented a significantly elevated TNF-α level and body temperature, suggesting that *ApoE-*ε*4* is related to immune response enhancement *in vivo* (Gale et al., 2014). Furthermore, ApoE3 was proved to be capable of inhibiting TLR4-mediated macrophage activation (Zhu et al., 2010). In AD, *ApoE-*ε*4* gene dose has been known as a strong risk factor for LOAD (Corder et al., 1993). *ApoE-*ε*4* seems to play the detrimental effect in AD *via TLR4*-dependent way. It is reported that *ApoE-*ε*4* non-carriers could modify the risk of LOAD caused by sequence variants of *TLR4* (Chen et al., 2012). In the CNS, ApoE is not only a lipid-transport molecule but also a ligand for TREM2 (Yeh et al., 2016). It shows high affinity for TREM2 and facilitates the phagocytosis of apoptotic neurons (Atagi et al., 2015), leading to the suppression of homeostatic microglia. Deletion of the *TREM2* gene suppressed ApoE pathway-mediated phagocytosis of apoptotic neurons and restored the homeostatic microglia population in APP/PS1 mice (Krasemann et al., 2017). However, the direct link between ApoE and TLR4 in Aβ phagocytosis still remains vague and needs further exploration.

## High-Mobility Group Box Protein 1 and Toll-Like Receptor 4

In addition to its known ligands, TLR4 can be activated by harmful endogenous molecules such as high-mobility group box protein 1 (HMGB1) that promote inflammatory signaling pathways. The expression of HMGB1 was reported to be elevated in AD brains (Takata et al., 2003). HMGB1 is presumed to be released from dead neurons and thus signals their demise to neighboring cells during AD progression (Scaffidi et al., 2002; Takata et al., 2012). HMGB1 is a typical DAMP molecule that is known to interact with Aβ. Extracellular HMGB1 binds to Aβ42 monomers and prevents their oligomerization and inhibits the phagocytosis of Aβ42 by microglia by blocking Aβ42 internalization (Takata et al., 2003). Injection of anti-HMGB1 antibody reduced the levels of all Aβ species in the brain of 5 × FAD transgenic mice by stimulating phagocytosis (Fujita et al., 2016). Additionally, *HMGB1* gene silencing attenuated Aβ-induced inflammation in hippocampal neuron cultures, which was mediated by receptor for advanced glycation end products (RAGE) or TLR4 signaling (Nan et al., 2019). Intracerebroventricular administration of HMGB1 disrupted memory encoding in control mice and *TLR4* and *RAGE* gene knockout mice to similar degrees (Mazarati et al., 2011). Treatment of *RAGE* knockout mice with TLR4 antagonist blocked the amnesic effect of HMGB1, suggesting that the memory deficits induced by HMGB1 are mediated by TLR4 or RAGE (Mazarati et al., 2011), although the detailed mechanisms remain to be clarified. It was proposed that extracellular HMGB1 binds to TLR4, which is followed by the activation of TLR4/MAPK and phosphorylation of myristoylated alanine-rich c-kinase substrate (MARCKS) at Ser46; this induces neurite degeneration, leading to impaired memory function (Fujita et al., 2016). Collectively, these results demonstrate that HMGB1 contributes to AD progression *via* TLR4 signaling.

## Toll-Like Receptor 4 as Therapeutic Target in Alzheimer Disease Treatment

Given its significant impact on the pathogenesis of AD, therapeutic strategies that target TLR4 are promising treatments for this disease. Several studies have demonstrated that inhibiting TLR4 blocks the progression of AD. Given that TLR4 signaling is not only involved in Aβ clearance but also promotes the release of neurotoxic cytokines during neuroinflammation in AD, the activation of TLR4 may have both beneficial and harmful effects in patients. In fact, TLR4 activation appears to be detrimental, as administration of LPS is widely used for experimental induction of an AD-like state that includes neuroinflammation and memory deficits (Anaeigoudari et al., 2016; Zakaria et al., 2017). Numerous compounds targeting TLR4 have been shown to alleviate cognitive impairment and AD-like pathology in animal models (Table 1). Aβ injection in animals induces memory impairment and cognitive dysfunction as well as microglia mobilization similar to that observed in AD, which is useful for investigating the anti-inflammatory mechanisms of potential therapies (McLarnon, 2014). Chemical compounds such as soybean isoflavones, hesperetin, chotosan, atorvastatin, and alpha-linoleic acid have been shown to alleviate memory dysfunction in Aβ42-injected rodents (Ding et al., 2011; Chen et al., 2016; Wang et al., 2018; Ikram et al., 2019; Ali et al., 2020). The mechanistic basis for these effects may be the suppression of TLR4 and downstream pro-inflammatory cytokines. In the APP/PS1 mouse model of cerebral amyloid deposition, reducing TLR4 levels improved cognitive function. Treatment with TAK-242, a specific inhibitor of TLR4, alleviated learning and memory dysfunction, reduced Aβ accumulation, and protected neurons against apoptosis in APP/PS1 mice (Cui et al., 2020); and baicalin exerted similar neuroprotective effects in this model via TLR4/NF-κB signaling (Jin et al., 2019). Gx-50, a compound extracted from Sichuan pepper, has demonstrated anti-inflammatory effects in the AD brain; the mechanism of action involves the suppression of TLR4 followed by reduced recruitment of MyD88 and TRAF6, which blocked the nuclear translocation of NF-κB and phosphorylation of MAPK (Shi et al., 2016). Thymoquinone and ethyl pyruvate prevented cognitive decline and inhibited the expression of TLR4 as well as Aβ deposition in rats with aluminum chloride (AlCl_3_)-induced AD (Abulfadl et al., 2018; Chavali et al., 2020).

**TABLE 1:** Summary of preclinical studies investigating the efficacy of therapeutics targeting TLR4 for the treatment of AD-like pathology.

**Intervention (References)**	**Animal model**	**Treatment regimen**	**Observations**
Monophosphoryl lipid A (Michaud et al., 2013)	APP/PS1 transgenic mouse	50 μg once a week for 12 weeks, IP	• Induced a low inflammatory response while triggering a strong phagocytosis of Aβ in mice • Improved AD-related pathology and enhanced memory function in APP/PS1 mice
Thymoquinone (Abulfadl et al., 2018)	Rat model of AD induced by AlCl_3_ (10 mg/kg/day for 42 days, IP) and D-galactose (60 mg/kg/day for 42 days, IP)	10, 20, and 40 mg/kg/day for 14 days, IG	• Alleviated cognitive impairment in AD rats • Reduced Aβ deposition • Reduced TNF-α and IL-1β levels • Decreased expression of TLR4, MyD88, TRIF, and downstream effectors including NF-κB and IRF3
Soybean isoflavone (Ding et al., 2011)	Rat model of AD induced by Aβ42 (20 μg/200 μl)	80 mg/kg/day for 14 days, IG	• Improved learning and memory in rats • Reduced production of pro-inflammatory cytokines IL-1β and TNF-α • Reversed Aβ42-induced upregulation of TLR4 and nuclear translocation of NF-κB p65
Hesperetin (Ikram et al., 2019)	Mouse model of AD induced by Aβ42 (5 μg, ICV)	50 mg/kg for 6 weeks, ICV	• Reduced lipid peroxidation and reactive oxygen species production and increased Nrf2/HO-1 expression in response to oxidative stress in the brain • Reversed Aβ-induced microglia activation and reduced expression of APP, BACE-1, and Aβ • Attenuated expression of TLR4, p-NF-κB, TNF-α, and IL-1β and proapoptotic proteins such as Bax, Caspase-3, and PARP-1 in neurons • Increased levels of synaptic markers including syntaxin, SNAP-25, PSD-95, Syp, and SNAP-23 Alleviated memory dysfunction
Chotosan (Chen et al., 2016)	Mouse model of AD induced by Aβ42 (410 pM)	750 or 375 mg/kg/day for 3 weeks, IG	• Alleviated memory and cognitive deficits • Attenuated upregulation of TLR4 and NF-κB p65 as well as that of pro-inflammatory cytokines TNF-α and IL-1β • Inhibited neuronal apoptosis, as evidenced by an increase in Bcl-2/Bax ratio and a decrease in the level of proapoptotic protein Caspase-3
Ethyl pyruvate (Chavali et al., 2020)	Rat model of AD induced by AlCl_3_ (50 mg/kg/day for 28 days, IP)	50, 100, and 200 mg/kg/day, IG	• Alleviated cognitive impairment • Reduced oxidative stress as assessed by decreased MDA, nitrite, and SOD level and increased catalase and glutathione levels • Decreased expression of TLR4 • Ameliorated deposition of amyloid and neurofibrillary tangles
Atorvastatin (Wang et al., 2018)	Rat model of AD induced by Aβ42 (10 μl, ICV)	5 or 10 mg/kg from 3 weeks before to 6 days after injection of Aβ42, IG	• Alleviated cognitive impairment in rats • Attenuated microglia and astrocyte activation • Suppressed Aβ42-induced apoptosis • Reduced levels of TLR4 and TRAF6 and inhibited NF-κB nuclear translocation
Gx-50 (Shi et al., 2016)	APP transgenic mouse	1 mg/kg for 2 months, IP	• Suppressed microglia activation and expression of IL-1β, iNOS, and COX2 • Blocked Aβ-induced phosphorylation of IκB and NF-κB nuclear translocation • Decreased levels of TLR4, MyD88, and TRAF6 • Inhibited MAPK activation
Alpha linoleic acid (Ali et al., 2020)	Mouse model of AD induced by Aβ42 (5 μl, ICV)	60 mg/kg for 6 weeks, PO	• Attenuated Aβ42-induced activation of TLR4 and reduced GFAP and Iba-1 levels in the frontal cortex and hippocampus • Reduced the expression of p-JNK, p-NF-κB p65 (Ser536), and TNF • Enhanced the expression of APP, BACE-1, and Aβ42 in the brain • Reversed Aβ42-induced cell apoptosis • Increased the expression of synaptic markers (SNAP-23 and PSD-95) • Improved cognitive function
TAK-242 (Cui et al., 2020)	APP/PS1 transgenic mouse	2 mg/kg/day for 28 days, IP	• Promoted M1 microglia switching to an M2 phenotype • Reduced plaque load • Improved cognitive performance • Suppressed inflammatory response by inhibiting MyD88/NF-κB-p65 and NLRP3
Baicalin (Jin et al., 2019)	APP/PS1 transgenic mouse	103 mg/kg/day for 33 days, IG	• Alleviated deficits in learning and cognition • Blocked neuronal apoptosis • Inhibited microglia activation and production of pro-inflammatory cytokines including IL-1β, IL-18, and iNOS • Suppressed TLR4/NF-κB signaling and activation of the NLRP3 inflammasome

*Aβ, amyloid beta; AD, Alzheimer disease; AlCl_3_, aluminum chloride; APP, amyloid precursor protein; BACE-1, beta-amyloid–cleaving enzyme 1; Bax, B cell lymphoma 2-activated X protein; Bcl-2, B cell lymphoma 2; COX2, cyclooxygenase 2; GFAP, glial fibrillary acidic protein; HO-1, heme oxygenase 1; Iba-1, ionized calcium-binding adaptor molecule 1; ICV, intracerebroventricular; IG, intragastric; iNOS, inducible nitric oxide synthase; IL, interleukin; IP, intraperitoneal; IRF3, interferon-regulatory factor 3; LPO, lipid peroxidation; MAPK, mitogen-activated protein kinase; MDA, malondialdehyde; MyD88, myeloid differentiation primary response protein 88; NF-κB, nuclear factor kappa B; NLRP3, nucleotide-binding oligomerization domain, leucine- rich repeat, and pyrin domain-containing 3; Nrf2, nuclear factor erythroid 2-related factor 2; PARP, poly(ADP-ribose) polymerase; p-JNK, phosphorylated c-Jun N-terminal kinase; p-NF-κB, phosphorylated nuclear factor kappa B; PO, peroral; PS1, presenilin 1; PSD-95, postsynaptic density 95; SNAP, synaptosomal-associated protein; ROS, reactive oxygen species; SOD, superoxide dismutase, Syp, synaptophysin; TLR4, Toll-like receptor 4; TNF-α, tumor necrosis factor alpha; TRAF6, tumor necrosis factor receptor-associated factor 6; TRIF, TIR domain-containing adaptor protein-inducing interferon-β.*

## Discussion and Conclusion

Despite enormous research efforts, to date, there are no effective treatments for slowing or reversing the progression of AD. Novel therapeutics are therefore urgently needed. In this review, we summarized the key evidence for the involvement of TLR4 signaling in AD pathogenesis. We also described the mechanisms of action of TLR4 in AD progression. Several polymorphisms in the *TLR4* gene have been identified that are linked to AD, and TLR4-dependent mechanisms were shown to be essential for neuroinflammatory responses in AD. In animal models, a number of drugs and other compounds have been shown to alleviate disease symptoms mainly by inhibiting TLR4 signaling, microglia activation, and downstream pro-inflammatory cytokine production, thereby reducing oxidative stress and neuronal apoptosis and ultimately improving learning and cognitive functioning.

Although therapeutic targeting of TLR4 in animal models of AD or neuroinflammation has yielded promising results, translating this approach to clinical practice is not yet feasible. The immune system imbalance in AD is complex, with multiple interacting factors influencing neuroinflammation. Thus, a single mediator of inflammation may not be exclusively harmful or beneficial. In the future, it is important to explore the mechanisms between TLR4 and other receptors or proteins in order to determine the most effective therapeutic strategy for the treatment of AD.

## Author Contributions

YZ, YC, CX, and HZ drafted the manuscript. CL revised the manuscript. All authors have commented on and approved the final version of the manuscript for publication.

## Conflict of Interest

The authors declare that the research was conducted in the absence of any commercial or financial relationships that could be construed as a potential conflict of interest.

## References

[B1] Abulfadl Y. S. El-Maraghy N. N. Ahmed A. E. Nofal S. Abdel-Mottaleb Y. Badary O. A. (2018). Thymoquinone alleviates the experimentally induced Alzheimer’s disease inflammation by modulation of TLRs signaling. *Hum. Exper. Toxicol.* 37 1092–1104. 10.1177/0960327118755256 29405769

[B2] Akira S. Takeda K. (2004). Toll-like receptor signalling. *Nat. Rev. Immunol.* 4 499–511. 10.1038/nri1391 15229469

[B3] Akira S. Uematsu S. Takeuchi O. (2006). Pathogen recognition and innate immunity. *Cell* 124 783–801. 10.1016/j.cell.2006.02.015 16497588

[B4] Ali W. Ikram M. Park H. Y. Jo M. G. Ullah R. Ahmad S. (2020). Oral administration of alpha linoleic acid rescues Aβ-induced glia-mediated neuroinflammation and cognitive dysfunction in C57BL/6N mice. *Cells* 9:667. 10.3390/cells9030667 32182943 PMC7140708

[B5] Alzheimer’s Association (2019). 2019 Alzheimer’s disease facts and figures. *Alzheimer Dement.* 15 321–387.

[B6] Anaeigoudari A. Soukhtanloo M. Reisi P. Beheshti F. Hosseini M. (2016). Inducible nitric oxide inhibitor aminoguanidine, ameliorates deleterious effects of lipopolysaccharide on memory and long term potentiation in rat. *Life Sci.* 158 22–30. 10.1016/j.lfs.2016.06.019 27341994

[B7] Andreone B. J. Przybyla L. Llapashtica C. Rana A. Davis S. S. van Lengerich B. (2020). Alzheimer’s-associated PLCγ2 is a signaling node required for both TREM2 function and the inflammatory response in human microglia. *Nat. Neurosci.* 23 927–938. 10.1038/s41593-020-0650-6 32514138

[B8] Atagi Y. Liu C. C. Painter M. M. Chen X. F. Verbeeck C. Zheng H. (2015). Apolipoprotein E is a ligand for triggering receptor expressed on myeloid cells 2 (TREM2). *J. Biol. Chem.* 290 26043–26050. 10.1074/jbc.M115.679043 26374899 PMC4646257

[B9] Balducci C. Frasca A. Zotti M. La Vitola P. Mhillaj E. Grigoli E. (2017). Toll-like receptor 4-dependent glial cell activation mediates the impairment in memory establishment induced by β-amyloid oligomers in an acute mouse model of Alzheimer’s disease. *Brain Behav. Immun.* 60 188–197. 27751869 10.1016/j.bbi.2016.10.012

[B10] Bertram L. Lill C. M. Tanzi R. E. (2010). The genetics of Alzheimer disease: back to the future. *Neuron* 68 270–281. 10.1016/j.neuron.2010.10.013 20955934

[B11] Bowman C. C. Rasley A. Tranguch S. L. Marriott I. (2003). Cultured astrocytes express toll-like receptors for bacterial products. *Glia* 43 281–291. 10.1002/glia.10256 12898707

[B12] Bsibsi M. Ravid R. Gveric D. van Noort J. M. (2002). Broad expression of Toll-like receptors in the human central nervous system. *J. Neuropathol. Exper. Neurol.* 61 1013–1021. 12430718 10.1093/jnen/61.11.1013

[B13] Cacabelos R. Barquero M. García P. Alvarez X. A. Varela de Seijas E. (1991). Cerebrospinal fluid interleukin-1 beta (IL-1 beta) in Alzheimer’s disease and neurological disorders. *Methods Find. Exper. Clin. Pharmacol.* 13 455–458. 1784142

[B14] Castellani R. J. Perry G. (2012). Pathogenesis and disease-modifying therapy in Alzheimer’s disease: the flat line of progress. *Archiv. Med. Res.* 43 694–698. 10.1016/j.arcmed.2012.09.009 23085451

[B15] Chavali V. D. Agarwal M. Vyas V. K. Saxena B. (2020). Neuroprotective effects of ethyl Pyruvate against aluminum chloride-induced Alzheimer’s disease in rats via inhibiting toll-like receptor 4. *J. Mol. Neurosci.* 70 836–850. 10.1007/s12031-020-01489-9 32030557

[B16] Chen L. Hu L. Zhao J. Hong H. Feng F. Qu W. (2016). Chotosan improves Aβ1-42-induced cognitive impairment and neuroinflammatory and apoptotic responses through the inhibition of TLR-4/NF-κB signaling in mice. *J. Ethnopharmacol.* 191 398–407. 10.1016/j.jep.2016.03.038 26994819

[B17] Chen Y. C. Yip P. K. Huang Y. L. Sun Y. Wen L. L. Chu Y. M. (2012). Sequence variants of toll like receptor 4 and late-onset Alzheimer’s disease. *PLoS One* 7:e50771. 10.1371/journal.pone.0050771 23272070 PMC3525588

[B18] Corder E. H. Saunders A. M. Strittmatter W. J. Schmechel D. E. Gaskell P. C. Small G. W. (1993). Gene dose of apolipoprotein E type 4 allele and the risk of Alzheimer’s disease in late onset families. *Science* 261 921–923. 10.1126/science.8346443 8346443

[B19] Cui W. Sun C. Ma Y. Wang S. Wang X. Zhang Y. (2020). Inhibition of TLR4 induces M2 Microglial polarization and provides neuroprotection via the NLRP3 inflammasome in Alzheimer’s disease. *Front. Neurosci.* 14:444. 10.3389/fnins.2020.00444 32508567 PMC7251077

[B20] Ding B. Ma W. He L. Zhou X. Yuan L. Yu H. (2011). Soybean isoflavone alleviates β-amyloid 1-42 induced inflammatory response to improve learning and memory ability by down regulation of Toll-like receptor 4 expression and nuclear factor-κB activity in rats. *Intern. J. Dev. Neurosci.* 29 537–542. 10.1016/j.ijdevneu.2011.04.002 21515354

[B21] Doyle S. E. O’Connell R. M. Miranda G. A. Vaidya S. A. Chow E. K. Liu P. T. (2004). Toll-like receptors induce a phagocytic gene program through p38. *J. Exper. Med.* 199 81–90. 10.1084/jem.20031237 14699082 PMC1887723

[B22] Fujikura M. Iwahara N. Hisahara S. Kawamata J. Matsumura A. Yokokawa K. (2019). CD14 and toll-like receptor 4 promote Fibrillar Aβ42 uptake by microglia through A clathrin-mediated pathway. *J. Alzheimer Dis.* 68 323–337. 10.3233/jad-180904 30775984

[B23] Fujita K. Motoki K. Tagawa K. Chen X. Hama H. Nakajima K. (2016). HMGB1, a pathogenic molecule that induces neurite degeneration via TLR4-MARCKS, is a potential therapeutic target for Alzheimer’s disease. *Sci. Rep.* 6:31895. 10.1038/srep31895 27557632 PMC4997258

[B24] Gale S. C. Gao L. Mikacenic C. Coyle S. M. Rafaels N. Murray Dudenkov T. (2014). APOε4 is associated with enhanced in vivo innate immune responses in human subjects. *J. Allergy Clin. Immunol.* 134 127–134. 10.1016/j.jaci.2014.01.032 24655576 PMC4125509

[B25] Go M. Kou J. Lim J. E. Yang J. Fukuchi K. I. (2016). Microglial response to LPS increases in wild-type mice during aging but diminishes in an Alzheimer’s mouse model: implication of TLR4 signaling in disease progression. *Biochem. Biophys. Res. Commun.* 479 331–337. 10.1016/j.bbrc.2016.09.073 27641666 PMC5048480

[B26] Grammas P. Ovase R. (2001). Inflammatory factors are elevated in brain microvessels in Alzheimer’s disease. *Neurobiol. Aging* 22 837–842. 10.1016/s0197-4580(01)00276-7 11754990

[B27] Guerreiro R. Wojtas A. Bras J. Carrasquillo M. Rogaeva E. Majounie E. (2013). TREM2 variants in Alzheimer’s disease. *N. Engl. J. Med.* 368 117–127. 10.1056/NEJMoa1211851 23150934 PMC3631573

[B28] Hansen D. V. Hanson J. E. Sheng M. (2018). Microglia in Alzheimer’s disease. *J. Cell Biol.* 217 459–472. 10.1083/jcb.201709069 29196460 PMC5800817

[B29] Hatters D. M. Peters-Libeu C. A. Weisgraber K. H. (2006). Apolipoprotein E structure: insights into function. *Trends Biochem. Sci.* 31 445–454. 10.1016/j.tibs.2006.06.008 16820298

[B30] Ikram M. Muhammad T. Rehman S. U. Khan A. Jo M. G. Ali T. (2019). Hesperetin confers neuroprotection by regulating Nrf2/TLR4/NF-κB signaling in an Aβ mouse model. *Mol. Neurobiol.* 56 6293–6309. 10.1007/s12035-019-1512-7 30756299

[B31] Ito H. Hamerman J. A. (2012). TREM-2, triggering receptor expressed on myeloid cell-2, negatively regulates TLR responses in dendritic cells. *Eur. J. Immunol.* 42 176–185. 10.1002/eji.201141679 21956652 PMC3444819

[B32] Jevtic S. Sengar A. S. Salter M. W. McLaurin J. (2017). The role of the immune system in Alzheimer disease: etiology and treatment. *Age. Res. Rev.* 40 84–94. 10.1016/j.arr.2017.08.005 28941639

[B33] Jin J. J. Kim H. D. Maxwell J. A. Li L. Fukuchi K. (2008). Toll-like receptor 4-dependent upregulation of cytokines in a transgenic mouse model of Alzheimer’s disease. *J. Neuroinflamm.* 5:23. 10.1186/1742-2094-5-23 18510752 PMC2430555

[B34] Jin X. Liu M. Y. Zhang D. F. Zhong X. Du K. Qian P. (2019). Baicalin mitigates cognitive impairment and protects neurons from microglia-mediated neuroinflammation via suppressing NLRP3 inflammasomes and TLR4/NF-κB signaling pathway. *CNS Neurosci. Therap.* 25 575–590. 10.1111/cns.13086 30676698 PMC6488900

[B35] Karch C. M. Goate A. M. (2015). Alzheimer’s disease risk genes and mechanisms of disease pathogenesis. *Biolo. Psychiatry* 77 43–51. 10.1016/j.biopsych.2014.05.006 24951455 PMC4234692

[B36] Kawai T. Akira S. (2007). TLR signaling. *Semin. Immunol.* 19 24–32. 10.1016/j.smim.2006.12.004 17275323

[B37] Kawasaki T. Kawai T. (2014). Toll-like receptor signaling pathways. *Front. Immunol.* 5:461. 10.3389/fimmu.2014.00461 25309543 PMC4174766

[B38] Keren-Shaul H. Spinrad A. Weiner A. Matcovitch-Natan O. Dvir-Szternfeld R. Ulland T. K. (2017). A unique microglia type associated with restricting development of Alzheimer’s disease. *Cell* 169 1276–1290. 10.1016/j.cell.2017.05.018 28602351

[B39] Kong L. Ge B. X. (2008). MyD88-independent activation of a novel actin-Cdc42/Rac pathway is required for Toll-like receptor-stimulated phagocytosis. *Cell Res.* 18 745–755. 10.1038/cr.2008.65 18542102

[B40] Krasemann S. Madore C. Cialic R. Baufeld C. Calcagno N. El Fatimy R. (2017). The TREM2-apoe pathway drives the transcriptional phenotype of dysfunctional microglia in neurodegenerative diseases. *Immunity* 47 566–581.e9. 10.1016/j.immuni.2017.08.008 28930663 PMC5719893

[B41] Li X. Melief E. Postupna N. Montine K. S. Keene C. D. Montine T. J. (2015). Prostaglandin E2 receptor subtype 2 regulation of scavenger receptor CD36 modulates microglial Aβ42 phagocytosis. *Am. J. Pathol.* 185 230–239. 10.1016/j.ajpath.2014.09.016 25452117 PMC4278245

[B42] Long H. Zhong G. Wang C. Zhang J. Zhang Y. Luo J. (2019). TREM2 attenuates Aβ1-42-mediated neuroinflammation in BV-2 cells by downregulating TLR signaling. *Neurochem. Res.* 44 1830–1839. 10.1007/s11064-019-02817-1 31134514

[B43] Mazarati A. Maroso M. Iori V. Vezzani A. Carli M. (2011). High-mobility group box-1 impairs memory in mice through both toll-like receptor 4 and receptor for advanced glycation end products. *Exper. Neurol.* 232 143–148. 10.1016/j.expneurol.2011.08.012 21884699 PMC3202022

[B44] McGeer P. L. Itagaki S. Tago H. McGeer E. G. (1987). Reactive microglia in patients with senile dementia of the Alzheimer type are positive for the histocompatibility glycoprotein HLA-DR. *Neurosc. Lett.* 79 195–200. 10.1016/0304-3940(87)90696-3 3670729

[B45] McLarnon J. G. (2014). Correlated inflammatory responses and neurodegeneration in peptide-injected animal models of Alzheimer’s disease. *Biomed. Res. Intern.* 2014:923670. 10.1155/2014/923670 24822221 PMC4005142

[B46] Michaud J. P. Hallé M. Lampron A. Thériault P. Préfontaine P. Filali M. (2013). Toll-like receptor 4 stimulation with the detoxified Ligand Monophosphoryl lipid A improves Alzheimer’s disease-related pathology. *Proc. Natl. Acad. Sci. U.S.A.* 110 1941–1946. 10.1073/pnas.1215165110 23322736 PMC3562771

[B47] Minoretti P. Gazzaruso C. Vito C. D. Emanuele E. Bianchi M. Coen E. (2006). Effect of the functional toll-like receptor 4 Asp299Gly polymorphism on susceptibility to late-onset Alzheimer’s disease. *Neurosci. Lett.* 391 147–149. 10.1016/j.neulet.2005.08.047 16157451

[B48] Miron J. Picard C. Lafaille-Magnan M. Savard M. Labonté A. Breitner J. (2019). Association of TLR4 with Alzheimer’s disease risk and presymptomatic biomarkers of inflammation. *Alzheimers Dement.* 15 951–960. 10.1016/j.jalz.2019.03.012 31175027

[B49] Moresco E. M. LaVine D. Beutler B. (2011). Toll-like receptors. *Curr. Biol.* 21 R488–R493. 10.1016/j.cub.2011.05.039 21741580

[B50] Nan K. Han Y. Fang Q. Huang C. Yu L. Ge W. (2019). HMGB1 gene silencing inhibits neuroinflammation via down-regulation of NF-κB signaling in primary hippocampal neurons induced by Aβ(25-35). *Intern. Immunopharmacol.* 67 294–301. 10.1016/j.intimp.2018.12.027 30572254

[B51] Olson J. K. Miller S. D. (2004). Microglia initiate central nervous system innate and adaptive immune responses through multiple TLRs. *J. Immunol.* 173 3916–3924. 10.4049/jimmunol.173.6.3916 15356140

[B52] Pourbadie H. G. Sayyah M. Khoshkholgh-Sima B. Choopani S. Nategh M. Motamedi F. (2018). Early minor stimulation of microglial TLR2 and TLR4 receptors attenuates Alzheimer’s disease-related cognitive deficit in rats: behavioral, molecular, and electrophysiological evidence. *Neurobiol. Aging* 70 203–216. 10.1016/j.neurobiolaging.2018.06.020 30031930

[B53] Price B. R. Sudduth T. L. Weekman E. M. Johnson S. Hawthorne D. Woolums A. (2020). Therapeutic Trem2 activation ameliorates amyloid-beta deposition and improves cognition in the 5XFAD model of amyloid deposition. *J. Neuroinflamm.* 17:238 10.1186/s12974-020-01915-0 PMC7427742 32795308

[B54] Querfurth H. W. LaFerla F. M. (2010). Alzheimer’s disease. *N. Engl. J. Med.* 362 329–344. 10.1056/NEJMra0909142 20107219

[B55] Raetz C. R. H. Whitfield C. (2002). Lipopolysaccharide endotoxins. *Annu. Rev. Biochem.* 71 635–700. 10.1146/annurev.biochem.71.110601.135414 12045108 PMC2569852

[B56] Scaffidi P. Misteli T. Bianchi M. E. (2002). Release of chromatin protein HMGB1 by necrotic cells triggers inflammation. *Nature* 418 191–195. 10.1038/nature00858 12110890

[B57] Shi S. Liang D. Chen Y. Xie Y. Wang Y. Wang L. (2016). Gx-50 reduces β-amyloid-induced TNF-α, IL-1β, NO, and PGE2 expression and inhibits NF-κB signaling in a mouse model of Alzheimer’s disease. *Eur. J. Immunol.* 46 665–676. 10.1002/eji.201545855 26643273

[B58] Shi Y. Holtzman D. M. (2018). Interplay between innate immunity and Alzheimer disease: APOE and TREM2 in the spotlight. *Nat. Rev. Immunol.* 18 759–772. 10.1038/s41577-018-0051-1 30140051 PMC6425488

[B59] Sims R. van der Lee S. J. Naj A. C. Bellenguez C. Badarinarayan N. Jakobsdottir J. (2017). Rare coding variants in PLCG2, ABI3, and TREM2 implicate microglial-mediated innate immunity in Alzheimer’s disease. *Nat. Genet.* 49 1373–1384. 10.1038/ng.3916 28714976 PMC5669039

[B60] Song M. Jin J. Lim J. E. Kou J. Pattanayak A. Rehman J. A. (2011). TLR4 mutation reduces microglial activation, increases Aβ deposits and exacerbates cognitive deficits in a mouse model of Alzheimer’s disease. *J. Neuroinflamm.* 8:92. 10.1186/1742-2094-8-92 21827663 PMC3169468

[B61] Su F. Bai F. Zhou H. Zhang Z. (2016). Microglial toll-like receptors and Alzheimer’s disease. *Brain Behav. Immun.* 52 187–198. 10.1016/j.bbi.2015.10.010 26526648

[B62] Tahara K. Kim H. D. Jin J. J. Maxwell J. A. Li L. Fukuchi K. (2006). Role of toll-like receptor signalling in Abeta uptake and clearance. *Brain* 129(Pt 11), 3006–3019. 10.1093/brain/awl249 16984903 PMC2445613

[B63] Takata K. Kitamura Y. Kakimura J. Shibagaki K. Tsuchiya D. Taniguchi T. (2003). Role of high mobility group protein-1 (HMG1) in amyloid-beta homeostasis. *Biochem. Biophys. Res. Commun.* 301 699–703. 10.1016/s0006-291x(03)00024-x 12565837

[B64] Takata K. Takada T. Ito A. Asai M. Tawa M. Saito Y. (2012). Microglial amyloid-β1-40 phagocytosis dysfunction is caused by high-mobility group box protein-1: implications for the pathological progression of Alzheimer’s disease. *Intern. J. Alzheimer Dis.* 2012:685739. 10.1155/2012/685739 22645697 PMC3357001

[B65] Walter S. Letiembre M. Liu Y. Heine H. Penke B. Hao W. (2007). Role of the toll-like receptor 4 in neuroinflammation in Alzheimer’s disease. *Cell. Physiol. Biochem.* 20 947–956. 10.1159/000110455 17982277

[B66] Wang L. Z. Yu J. T. Miao D. Wu Z. C. Zong Y. Wen C. Q. (2011). Genetic association of TLR4/11367 polymorphism with late-onset Alzheimer’s disease in a Han Chinese population. *Brain Res.* 1381 202–207. 10.1016/j.brainres.2011.01.007 21236243

[B67] Wang S. Zhang X. Zhai L. Sheng X. Zheng W. Chu H. (2018). Atorvastatin attenuates cognitive deficits and neuroinflammation induced by Aβ(1-42) involving modulation of TLR4/TRAF6/NF-κB pathway. *J. Mol. Neurosci.* 64 363–373. 10.1007/s12031-018-1032-3 29417448

[B68] Yeh F. L. Wang Y. Tom I. Gonzalez L. C. Sheng M. (2016). TREM2 binds to Apolipoproteins, including APOE and CLU/APOJ, and thereby facilitates uptake of amyloid-Beta by microglia. *Neuron* 91 328–340. 10.1016/j.neuron.2016.06.015 27477018

[B69] Yousefi N. Sotoodehnejadnematalahi F. Heshmati-Fakhr N. Sayyah M. Hoseini M. Ghassemi S. (2019). Prestimulation of microglia through TLR4 pathway promotes interferon beta expression in a rat model of Alzheimer’s disease. *J. Mol. Neurosci.* 67 495–503. 10.1007/s12031-018-1249-1 30610591

[B70] Yu J. T. Miao D. Cui W. Z. Ou J. R. Tian Y. Wu Z. C. (2012). Common variants in toll-like receptor 4 confer susceptibility to Alzheimer’s disease in a Han Chinese population. *Curr. Alzheimer Res.* 9 458–466. 10.2174/156720512800492495 22272615

[B71] Zakaria R. Wan Yaacob W. M. Othman Z. Long I. Ahmad A. H. Al-Rahbi B. (2017). Lipopolysaccharide-induced memory impairment in rats: a model of Alzheimer’s disease. *Physiol. Res.* 66 553–565. 10.33549/physiolres.933480 28406691

[B72] Zhong L. Xu Y. Zhuo R. Wang T. Wang K. Huang R. (2019). Soluble TREM2 ameliorates pathological phenotypes by modulating microglial functions in an Alzheimer’s disease model. *Nat. Commun.* 10:1365. 10.1038/s41467-019-09118-9 30911003 PMC6433910

[B73] Zhou J. Yu W. Zhang M. Tian X. Li Y. Lü Y. (2019). Imbalance of Microglial TLR4/TREM2 in LPS-Treated APP/PS1 transgenic mice: a potential link between Alzheimer’s disease and systemic inflammation. *Neurochem. Res.* 44 1138–1151. 10.1007/s11064-019-02748-x 30756214

[B74] Zhu Y. Kodvawala A. Hui D. Y. (2010). Apolipoprotein E inhibits toll-like receptor (TLR)-3- and TLR-4-mediated macrophage activation through distinct mechanisms. *Biochem. J.* 428 47–54. 10.1042/bj20100016 20218969 PMC3050041

